# Paroxysmal Atrial Fibrillation in Liver Diseases: Epidemiology and Possible Pathophysiological Mechanisms

**DOI:** 10.3390/jcm15031156

**Published:** 2026-02-02

**Authors:** Lavinia Alice Bălăceanu, Cristiana Grigore, Beatrice Bălăceanu-Gurău, Cristian-Dorin Gurău, Ioana Valeria Grigorescu, Ion Dina

**Affiliations:** 1 Department of Medical Semiology, “Sf. Ioan” Clinical Emergency Hospital, “Carol Davila” University of Medicine and Pharmacy, 020021 Bucharest, Romania; alice.balaceanu@umfcd.ro (L.A.B.); cristiana.draganescu@drd.umfcd.ro (C.G.); ion.dina@umfcd.ro (I.D.); 2 Internal Medicine Clinic, “Sf. Ioan” Clinical Emergency Hospital, 042122 Bucharest, Romania; 3 “Carol Davila” University of Medicine and Pharmacy, 020021 Bucharest, Romania; ioana-valeria.grigorescu@rez.umfcd.ro; 4 Orthopedics and Traumatology Clinic, Clinical Emergency Hospital, “Carol Davila” University of Medicine and Pharmacy, 020021 Bucharest, Romania; 5 Clinical Department of Gastroenterology, “Sf. Ioan” Clinical Emergency Hospital, 042122 Bucharest, Romania

**Keywords:** metabolic dysfunction-associated steatotic liver disease, hepatic steatosis, cirrhosis, cardiometabolic risk, dyslipidemia, atrial cardiomyopathy

## Abstract

**Background:** Atrial fibrillation (AF) is frequently associated with cardiometabolic comorbidities, and increasing evidence suggests a close relationship between AF and liver disease, particularly metabolic dysfunction-associated steatotic liver disease (MASLD); however, the clinical patterns, hepatic phenotypes, and clinical implications of this association remain insufficiently characterized. Therefore, the aim of the present study was to characterize hepatic involvement in patients with paroxysmal AF by integrating a structured literature review with original clinical data. **Methods:** We performed a retrospective analysis of 253 patients admitted with paroxysmal AF between 2015 and 2025. Demographic data and associated diagnoses were collected with a specific focus on hepatic pathology. Patients were stratified according to the presence and type of liver disease, and descriptive statistics, bivariate analyses, and multivariate logistic regression were used to identify associations and independent predictors. **Results:** Liver disease was identified in 65.2% of patients, most commonly hepatic steatosis (46.2%), followed by liver cirrhosis or advanced liver disease (19.0%). Patients with liver disease had higher prevalences of type 2 diabetes mellitus, dyslipidemia, obesity, and alcohol consumption. Dyslipidemia (OR 4.51) and obesity (OR 2.54) were independent predictors of hepatic steatosis, whereas liver cirrhosis was inversely associated with age and serum lipid levels. **Conclusions:** Liver pathology is highly prevalent among patients with paroxysmal AF and is closely associated with adverse metabolic and clinical profiles. Recognition of distinct hepatic phenotypes may support improved risk stratification and multidisciplinary management in patients with AF.

## 1. Introduction

The prevalence of atrial fibrillation (AF) and atrial flutter has doubled worldwide between 1990 and 2019 [1]. The most pronounced increase has been reported in middle-income countries, ranging from 145.2% to 146.6%, whereas high-income countries experienced a lower increase of 67.8% during the same period [1].

Multiple clinical conditions and complex pathophysiological mechanisms contribute to the onset and maintenance of AF. Established risk factors include cardiovascular disease, hyperthyroidism, obesity, sleep apnea syndrome, chronic obstructive pulmonary disease, and alcohol consumption [2,3]. Additional factors include increased atrial pressure, inflammation, autonomic imbalance, and genetic predisposition [4]. Once initiated, AF is followed by atrial remodeling, characterized by both structural and electrophysiological alterations [4]. Progressive atrial cardiomyopathy involves fibrosis, hypertrophy, and consequently atrial dilation [4]. Younger patients with multiple first-degree relatives affected by AF may exhibit a genetic susceptibility, either monogenic or polygenic, although this subgroup represents only approximately 5% of all patients with AF [5].

Recently, the concept of atrial cardiomyopathy has gained increasing attention as a unifying framework for understanding the development and progression of AF. Atrial cardiomyopathy refers to a spectrum of structural, architectural, contractile, and electrophysiological abnormalities of the atrial myocardium that may precede, promote, or perpetuate AF, even in the absence of overt arrhythmia [6]. These alterations include atrial fibrosis, myocyte hypertrophy, impaired calcium handling, conduction heterogeneity, and reduced atrial compliance, ultimately creating a vulnerable substrate for AF initiation and maintenance [6]. Importantly, atrial cardiomyopathy is increasingly recognized as a dynamic and potentially reversible process driven by systemic conditions such as metabolic dysfunction, inflammation, neurohormonal activation, and organ crosstalk, rather than AF itself being the sole causal factor [6]. Recent evidence supports the role of atrial cardiomyopathy as a central determinant of AF risk, burden, and recurrence, highlighting its relevance for risk stratification and a mechanistic understanding of AF beyond traditional triggers and risk factors [6].

Within this framework, metabolic liver disease represents a prototypical systemic condition capable of promoting atrial cardiomyopathy through inflammatory, metabolic, and neurohormonal pathways. Metabolic dysfunction-associated steatotic liver disease (MASLD) has emerged as a significant risk factor for atrial fibrillation [7]. Low- and lower-middle-income countries carry the highest global burden of MASLD-related cardiometabolic complications, with an estimated prevalence of 533.65 million cases [8]. Between 2000 and 2019, the prevalence of AF and atrial flutter attributable to metabolic causes increased by 87% in these regions [9]. Beyond the classical components of metabolic syndrome—hypertension, obesity, dyslipidemia, and diabetes mellitus—insulin resistance and chronic low-grade inflammation play central roles in AF pathogenesis [7]. Insulin resistance promotes adipocyte inflammation, oxidative stress, impaired intracellular calcium handling, and structural atrial remodeling, leading to low-voltage areas and left atrial fibrosis [7]. Hyperinsulinemia not only facilitates AF onset but also contributes to disease progression and recurrence following catheter ablation [7]. Dyslipidemia further amplifies systemic inflammation, endothelial dysfunction, autonomic imbalance, and atrial electrical remodeling, thereby increasing AF susceptibility [7]. In addition, gut microbiota dysbiosis contributes to AF initiation through proinflammatory cytokine release and to AF progression via the modulation of autonomic nervous system activity [7].

Several studies have identified AF as the most frequent incidental cardiovascular condition associated with chronic liver disease, with reported prevalence rates of 30.2% in autoimmune liver disease, 25.6% in alcoholic liver disease, 20.8% in nonalcoholic fatty liver disease (NAFLD), 15.4% in chronic hepatitis B virus infection, and 16.8% in chronic hepatitis C virus infection [10]. NAFLD is frequently associated with AF through mechanisms involving atherosclerosis and systemic inflammation, independently of other metabolic syndrome components, including type 2 diabetes mellitus [11]. In recognition of the underlying metabolic dysfunction and its multisystem involvement, the term nonalcoholic fatty liver disease has been replaced by metabolic dysfunction-associated steatotic liver disease (MASLD) [12]. The diagnosis of MASLD requires evidence of hepatic steatosis—confirmed histologically, by imaging, or by validated biomarkers—together with at least one of the following criteria: obesity, type 2 diabetes mellitus, or the presence of at least two metabolic abnormalities associated with increased cardiovascular risk [12]. These metabolic abnormalities include blood pressure ≥130/85 mmHg or treated hypertension, low high-density lipoprotein cholesterol (HDL-C) levels, elevated triglycerides, prediabetes or increased insulin resistance, and elevated high-sensitivity C-reactive protein levels [12]. A recent meta-analysis showed that NAFLD significantly increases the risk of AF, particularly in Asian populations [13]. Systemic inflammation, oxidative stress, ectopic fat deposition in cardiac structures, autonomic dysfunction, and adverse cardiac remodeling collectively contribute to AF development through complex and interrelated mechanisms [13]. Proatherogenic lipid profiles and metabolic dysregulation are common pathophysiological pathways linking NAFLD and AF [14].

In this study, the nomenclature hepatic steatosis is used as a clinical term to describe fatty liver disease identified in routine practice, including MASLD and alcohol-related steatosis, whereas advanced liver disease refers to cirrhosis and its related complications, regardless of underlying etiology [14,15,16,17,18,19,20].

The aim of our study was twofold: to synthesize current evidence regarding the epidemiological and pathophysiological links between liver disease and AF, with particular emphasis on MASLD; and to evaluate, in a real-world cohort of patients with paroxysmal AF, the prevalence, clinical patterns, and metabolic correlates of hepatic pathology. By integrating a structured literature review with original clinical data, this study seeks to address the existing gap in the characterization of hepatic phenotypes in AF and their potential implications for risk stratification and multidisciplinary management.

## 2. Materials and Methods

### 2.1. Study Design

This retrospective study was conducted on a cohort of 253 patients diagnosed with paroxysmal AF, admitted to “Sf. Ioan” Clinical Emergency Hospital, Bucharest, Romania within the Departments of Internal Medicine and Gastroenterology between 1 January 2015 and 20 September 2025. Patient diagnoses were identified using both the Diagnosis-Related Group (DRG) system and free-text medical records, including primary and secondary diagnoses. In total, 5242 medical records were screened.

### 2.2. Data Collection

For each patient, demographic data (age at admission and biological sex) and associated diagnoses (comorbidities) were collected. Age was analyzed both as a continuous variable and categorically using clinically relevant thresholds (<65 years, 65–74 years, and ≥75 years), commonly applied in cardiovascular risk stratification and epidemiological studies [21,22].

More than 70 associated diagnoses were identified; for clarity of presentation, the 20 most prevalent comorbidities were selected for descriptive analysis. Liver pathology was defined based on clinical documentation and diagnostic coding and was categorized into hepatic steatosis and advanced liver disease/cirrhosis. The category hepatic steatosis included imaging- or clinically documented fatty liver disease, encompassing MASLD (formerly nonalcoholic fatty liver disease) and alcohol-related steatosis. The category advanced liver disease/cirrhosis included patients with established liver cirrhosis of any etiology (metabolic, alcoholic, viral, or other), as well as those with clinical or imaging evidence of portal hypertension, chronic liver failure, or primary or secondary hepatic malignancy.

### 2.3. Statistical Analysis

All statistical analyses were performed using SPSS software, version 21 (SPSS Inc., Chicago, IL, USA). Descriptive statistics were used to summarize the baseline characteristics. Continuous variables were expressed as the mean, standard deviation, and coefficient of variation, with median and quartiles additionally explored and visualized using box plots. Categorical variables were presented as absolute frequencies and percentages, including distributions by sex, age group, and comorbidity prevalence.

The distribution of continuous variables, particularly age (for the entire cohort and stratified by sex), was assessed using the Shapiro–Wilk and Kolmogorov–Smirnov tests to evaluate normality and justify the use of parametric statistical methods.

Group comparisons were performed to evaluate differences in comorbidity burden between patients with and without liver disease. The mean number of associated diagnoses was compared using the independent-samples *t*-test. Comparisons between categorical variables were performed using the chi-square test or Fisher’s exact test, as appropriate.

Bivariate associations between liver disease and selected metabolic or lifestyle-related comorbidities (including type 2 diabetes mellitus, dyslipidemia, obesity, and alcohol consumption) were assessed using crude risk ratios (RRs) to quantify differences in prevalence between groups.

To identify independent predictors of hepatic involvement and to control for potential confounding, multivariable logistic regression models were constructed. Based on clinical and pathophysiological considerations, two hepatic disease categories were defined a priori: (1) isolated hepatic steatosis, in the absence of liver cirrhosis, chronic liver failure, or hepatic malignancy; and (2) advanced liver disease, including liver cirrhosis, chronic liver failure, and/or hepatic malignancy, with or without concomitant steatosis. Separate regression models were developed for isolated hepatic steatosis and for advanced liver disease/cirrhosis.

Diagnoses and comorbidities were coded as binary variables (1 = present, 0 = absent) based on DRG coding and review of clinical documentation. Covariates were selected a priori according to clinical relevance and evidence from the literature and included age (expressed per 10-year increase), sex, type 2 diabetes mellitus, dyslipidemia, obesity, chronic kidney disease, and alcohol consumption, which was available only as a dichotomous variable (present/absent). Age and sex were retained in all models irrespective of statistical significance. No automated variable selection procedures (e.g., stepwise methods) were applied; all pre-specified covariates were entered simultaneously. Results are reported as adjusted odds ratios (ORs) with 95% confidence intervals (95% CI) and corresponding *p* values, with graphical presentation provided to enhance interpretability.

Model specification was intentionally parsimonious to minimize the risk of overfitting and was guided by the events-per-variable (EPV) framework. Within the study cohort, the outcome “isolated hepatic steatosis” comprised 110 events, whereas the outcome “liver cirrhosis” comprised 45 events. The final models included two predictors each, yielding EPV values of 55 and 22, respectively, which were well above the commonly accepted thresholds. Methodological studies indicate that EPV values between 5 and 9 are sufficient to limit bias and type I error in logistic regression when effect sizes are moderate to large [23,24].

In addition, a sensitivity analysis based on the fixed sample size (*n* = 253) indicated that the study had 80% statistical power (α = 0.05) to detect a minimum odds ratio of approximately 1.6 for predictors of hepatic steatosis and 2.3 (or a protective OR < 0.43) for predictors of cirrhosis, supporting adequate sensitivity for identifying moderate-to-large clinical effects.

A *p*-value < 0.05 was considered statistically significant for all analyses.

### 2.4. Literature Review

In parallel, a structured narrative literature review was conducted to contextualize the study findings and to identify relevant evidence regarding atrial fibrillation in the setting of liver and gastroenterological diseases, including both non-malignant and malignant conditions. Searches were performed in the Web of Science Core Collection, PubMed, Elsevier ScienceDirect, the Cochrane Database, Google Scholar, and international clinical guideline repositories.

The initial search was conducted in January 2025 and updated monthly through July 2025. The review was not intended as a systematic review, but as background and contextual support for study design and interpretation. Full-text original articles, narrative reviews, and case reports published in English between 1 January 2014 and 15 July 2025 were considered. Both Medical Subject Headings (MeSH) and free-text keywords (e.g., “atrial fibrillation”, “ascites”, “cirrhosis”, “gastroenterology”, “liver”) were used.

From the retrieved 2177 records, 97 publications were identified as relevant and informed the conceptual framework and discussion of the present study.

## 3. Results

### 3.1. Demographic Characteristics

**Figure 1:**
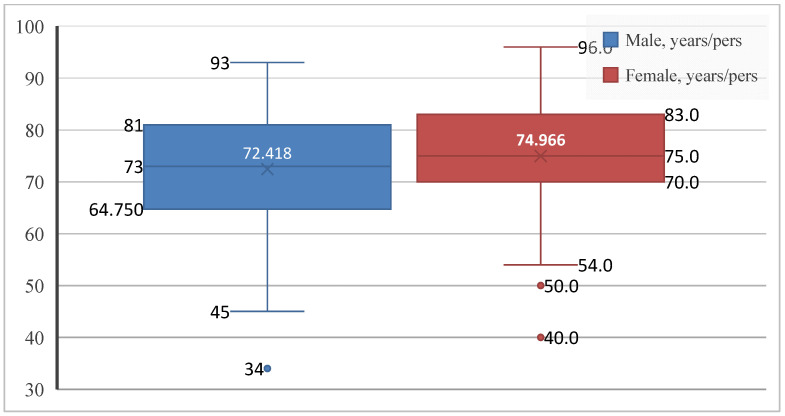
Age distribution by sex in patients with paroxysmal AF. Boxplots show the median, interquartile range, and extreme values.

Given the relevance of age and sex as key components of cardiovascular risk, the first step was to describe the demographic characteristics of the study population. Patients with paroxysmal AF ranged in age from 34 to 96 years. Of the 253 patients included, 134 were men (53.0%) and 119 were women (47.0%), showing an almost balanced sex distribution, with a slight predominance of male patients. The mean age of the overall cohort was 73.62 ± 10.46 years, in line with the established epidemiological profile of the disease. Women were slightly older than men (74.97 ± 9.57 vs. 72.42 ± 11.05 years), with greater age variability observed among male patients (Figure 1).

**Figure 2:**
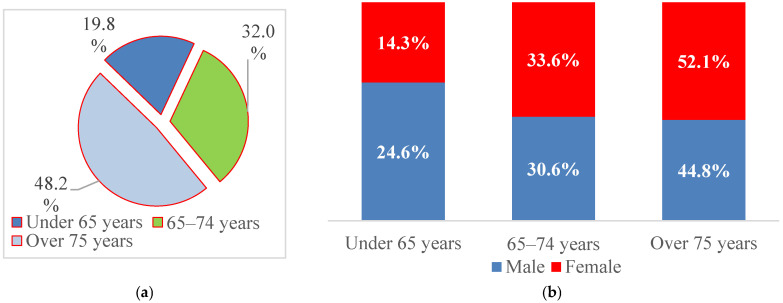
Structure of patients with paroxysmal AF by (**a**) age at admission and (**b**) sex.

For further analysis, patient age was categorized into three clinically relevant groups (<65 years, 65–74 years, and ≥75 years), reflecting the predominantly elderly distribution of AF and thresholds commonly used in cardiovascular risk stratification and epidemiological studies. Nearly half of the patients were aged ≥75 years (48.2%), 32.0% were between 65 and 74 years, and 19.8% were younger than 65 years (Figure 2). Men were more frequently represented in the <65-year group (24.6% vs. 14.3%), whereas women predominated among patients aged ≥75 years (52.1% vs. 44.8%), suggesting an earlier onset of AF in men and hospitalization at more advanced ages among women.

### 3.2. Comorbidity Burden

**Figure 3:**
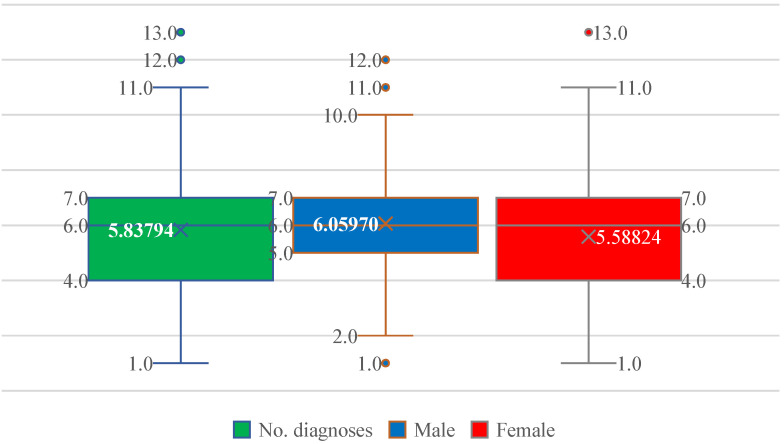
Distribution of the number of comorbid diagnoses (No. diagnoses) in patients with paroxysmal AF, shown for the entire cohort and stratified by sex. Boxplots display the median, interquartile range, and extreme values; the cross indicates the mean. The comorbidity burden is similar in men and women, with a median of six diagnoses and overlapping distributions.

Patients exhibited a substantial burden of associated diseases, ranging from 1 to 13 comorbidities. The median number of comorbid conditions was 6, with a mean of 5.84, reflecting a high degree of multimorbidity (Figure 3). The interquartile range spanned 4–7 diagnoses in both sexes, with similar mean values in men (6.06) and women (5.59), indicating comparable comorbidity loads (Figure 3).

More than half of the patients (52.6%) had 4–6 associated diagnoses, while 32.0% had 7–9 comorbidities and 4.0% had ≥10 diagnoses. Only 11.5% of patients presented with fewer than four comorbid conditions.

Patients with liver disease had a significantly higher mean number of associated diagnoses compared with patients without hepatic involvement (6.34 ± 2.06 vs. 4.90 ± 1.78 diagnoses; independent-samples *t*-test, *p* < 0.001), confirming a substantially greater multimorbidity burden in the liver disease group.

### 3.3. Spectrum of Comorbidities

**Figure 4:**
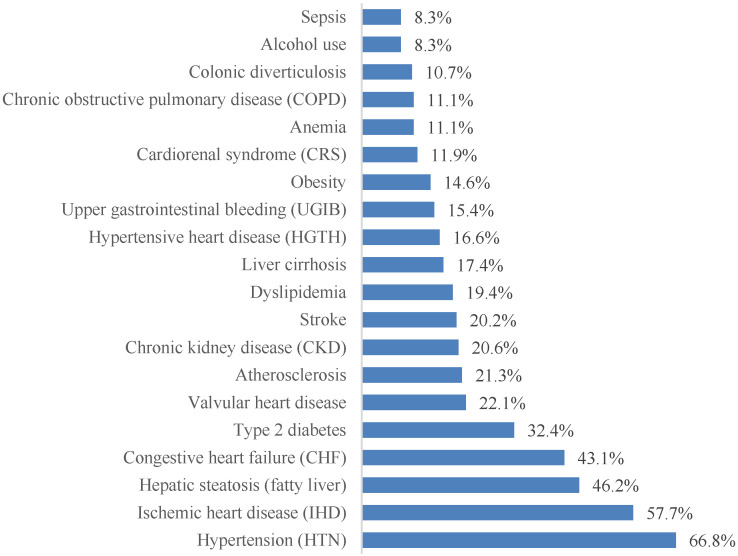
The most frequently encountered comorbidities in patients with paroxysmal AF, ordered by prevalence.

More than 70 distinct associated diagnoses were identified. For clarity, the 20 most prevalent conditions are presented in Figure 4. Cardiovascular pathology dominated the clinical profile, with arterial hypertension (66.8%), coronary artery disease (57.7%), and chronic heart failure (43.1%) being the most frequent comorbidities. Metabolic and hepatic conditions were also highly prevalent, including hepatic steatosis (46.2%) and type 2 diabetes mellitus (32.4%).

**Figure 5:**
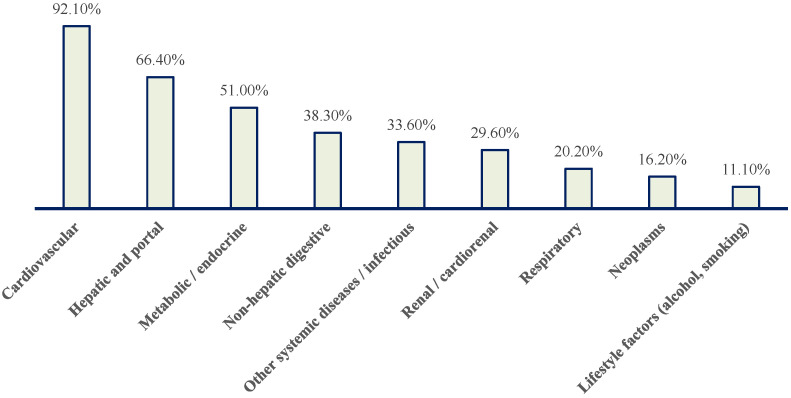
Distribution of patients with paroxysmal AF according to major pathology blocks. Cardiovascular conditions predominate, followed by hepatic and portal, metabolic/endocrine, non-hepatic digestive, renal/cardiorenal, respiratory, neoplastic, other systemic/infectious diseases, and lifestyle-related factors (alcohol use and smoking).

To synthesize the complex comorbidity spectrum, associated diagnoses were grouped into major pathology blocks (Figure 5 and Table 1).

Nearly all patients (92.1%) had at least one cardiovascular condition (Figure 5). Hepatic or portal pathology was present in 66.4% of patients, while metabolic/endocrine disorders affected 51.0% (Figure 5). Non-hepatic gastrointestinal diseases were observed in 38.3%, renal or cardiorenal involvement in 29.6%, respiratory pathology in 20.2%, and neoplastic disease in 16.2% (Figure 5). One third of patients had additional systemic or infectious conditions.

Overall, this distribution highlights a population characterized by advanced multimorbidity, with frequent overlap between cardiovascular disease, metabolic dysfunction, and liver pathology.

### 3.4. Liver Pathology in Patients with Paroxysmal AF

The overall prevalence of liver disease was similar in men and women (64.2% vs. 66.4%), with no statistically significant difference between sexes (χ^2^ test, *p* = 0.81). Therefore, subsequent analyses did not identify a sex-related effect on the presence of liver disease.

Liver disease was identified in 165 patients (65.2%), underscoring its central role in the comorbidity profile of paroxysmal AF. Hepatic steatosis was the most common diagnosis (46.2%), followed by liver cirrhosis (17.8%). Advanced liver disease—defined as cirrhosis, liver failure, and/or hepatic tumors—was present in 19.0% of patients, while isolated steatosis accounted for 43.5%.

Clinical manifestations of portal hypertension were observed in a relevant subgroup, with ascites present in 7.1% and esophageal varices in 6.3%, indicating an increased hemorrhagic risk.

Although the overall prevalence of liver disease did not differ by sex, descriptive analyses suggested that hepatic steatosis was more frequent among women, whereas cirrhosis was more prevalent among men. Liver pathology was most common in patients under 75 years of age, with a decreasing prevalence at very advanced ages, particularly for cirrhosis.

**Figure 6:**
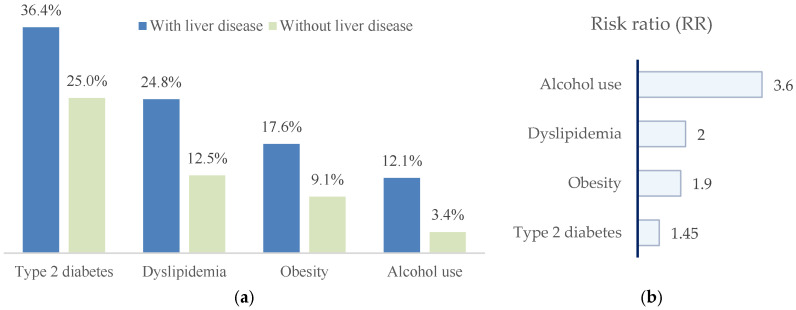
(**a**) Distribution of metabolic risk factors and alcohol consumption in patients with paroxysmal AF, stratified by the presence or absence of liver disease. Patients with liver disease show higher prevalences of type 2 diabetes mellitus, dyslipidemia, obesity, and alcohol use; (**b**) risk ratios (RRs), indicating the strength of association between each factor and liver disease.

Patients with liver disease exhibited a markedly less favorable metabolic and lifestyle profile. Compared with those without liver involvement, they showed higher prevalences of type 2 diabetes mellitus (36.4% vs. 25.0%), dyslipidemia (23.6% vs. 11.4%), obesity (17.6% vs. 9.1%), and alcohol consumption (11.5% vs. 2.3%) (Figure 6).

In univariate analyses, patients with liver disease showed higher prevalences of metabolic risk factors and alcohol consumption. Crude risk ratios were as follows: type 2 diabetes mellitus, RR = 1.45 (95% CI 0.96–2.20; *p* = 0.07); dyslipidemia, RR = 1.99 (95% CI 1.08–3.67; *p* = 0.02); obesity, RR = 1.93 (95% CI 0.92–4.05; *p* = 0.09); and alcohol consumption, RR = 3.56 (95% CI 1.09–11.64; *p* = 0.02). Accordingly, dyslipidemia and alcohol consumption were significantly associated with liver disease, whereas type 2 diabetes mellitus and obesity showed non-significant trends toward increased risk.

### 3.5. Independent Predictors and Hepatic Phenotypes

**Figure 7:**
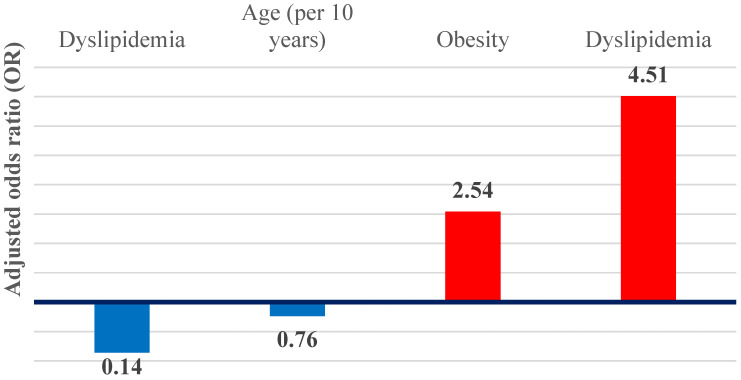
Graphical representation of the OR adjusted for independent predictors of hepatic steatosis and cirrhosis.

In multivariable logistic regression models, dyslipidemia and obesity remained independent predictors of hepatic steatosis (Figure 7). Dyslipidemia was associated with an approximately 4.5-fold increase in the odds of steatosis (adjusted OR 4.51; 95% CI 2.23–9.12; *p* < 0.001), while obesity was associated with an almost threefold increase (adjusted OR 2.54; 95% CI 1.15–5.65; *p* = 0.02). Together, these associations define a metabolic–steatotic hepatic phenotype.

In contrast, liver cirrhosis demonstrated inverse associations with metabolic factors. Dyslipidemia was significantly inversely associated with cirrhosis (adjusted OR 0.14; 95% CI 0.03–0.63; *p* = 0.01), while age showed a non-significant inverse trend when expressed per 10-year increase (adjusted OR 0.76; 95% CI 0.55–1.05; *p* = 0.09), suggestive of an advanced cirrhotic phenotype.

The multivariable models were intentionally specified in a parsimonious manner to ensure an adequate events-per-variable (EPV) ratio. Within the study cohort, there were 110 cases of isolated hepatic steatosis and 45 cases of liver cirrhosis; therefore, the inclusion of a limited number of predictors was deliberately chosen to minimize the risk of model overfitting.

Together, these results highlight the dual nature of liver involvement in paroxysmal AF and its close interplay with metabolic dysfunction and disease severity.

### 3.6. Serum Biomarker Profiles Across Hepatic Phenotypes

Serum biomarkers including hemoglobin, platelet count, albumin, AST, ALT, total bilirubin, alkaline phosphatase, cholesterol, triglycerides, and INR were compared across three hepatic phenotypes (no liver disease, hepatic steatosis, and advanced liver disease/cirrhosis). Continuous variables are reported as median [Q1–Q3], and between-group differences were assessed using the Kruskal–Wallis test. Significant global differences across hepatic phenotypes were observed for AST, albumin, total bilirubin, alkaline phosphatase, cholesterol, INR, platelet count, and hemoglobin (all *p* < 0.05), whereas ALT and triglyceride levels did not differ significantly between groups (Table 2).

These findings indicate that hepatic phenotypes are characterized by distinct biological signatures rather than merely diagnostic labels. Markers reflecting hepatic synthetic function and coagulation (albumin, INR), together with severity-associated parameters (bilirubin, platelet count, AST, ALP), clearly discriminated between groups. In contrast, isolated markers of hepatocellular injury, such as ALT, showed limited phenotyping value in this cohort.

The advanced/cirrhotic phenotype exhibited a biological profile consistent with more severe hepatic dysfunction, including higher INR and bilirubin levels, lower albumin concentrations and platelet counts, and higher AST and ALP values, particularly when compared with the steatotic phenotype. The distribution of anticoagulant therapy did not differ significantly across hepatic phenotypes.

Overall, these data support the presence of two hepatic phenotypes with distinct functional and systemic profiles in patients with paroxysmal atrial fibrillation.

## 4. Discussion

These findings support the presence of two distinct hepatic phenotypes in patients with AF (Figure 6). The metabolic–steatosis phenotype is characterized by obesity and dyslipidemia and reflects early-stage, metabolically driven liver disease. In contrast, the advanced cirrhotic phenotype is associated with lower lipid levels and younger age, consistent with impaired hepatic synthetic function and increased competitive mortality rather than metabolic protection.

Beyond common risk factors for AF, both MASLD and alcoholic liver disease have emerged as independent contributors to AF development, particularly in patients with type 2 diabetes mellitus [25]. In individuals with NAFLD, the risk of AF has been reported to increase by up to twofold, independently of established cardiovascular risk factors [26,27,28,29]. However, some authors debate that the association between NAFLD and AF becomes clinically relevant primarily in the context of metabolic syndrome, reflecting shared pathophysiological pathways underlying cardiovascular disease [30].

Evidence from population-based studies is heterogeneous. In the Framingham Heart Study, a longitudinal cohort with 12 years of follow-up, hepatic steatosis diagnosed by computed tomography was not significantly associated with incident AF [31]. In contrast, other studies suggest complex interactions between NAFLD and the cardiac conduction system, involving both structural and functional alterations [32,33]. NAFLD has been associated with cardiac remodeling, including left ventricular structural changes and left atrial enlargement [34]. Structural abnormalities such as type I left ventricular diastolic dysfunction and aortic valve sclerosis have also been linked to NAFLD, and impaired left ventricular filling may further predispose to AF [35].

Epicardial adipose tissue has been associated with incidental AF in multiple studies [36]. Excess epicardial and myocardial fat deposition, followed by structural and electrophysiological alterations, may represent a key mechanistic link between NAFLD and AF [37]. Atrial remodeling in this context is thought to be mediated by inflammation and oxidative stress, involving decreased adiponectin levels and increased proinflammatory cytokines such as interleukin-6 and tumor necrosis factor-α [38,39]. Insulin resistance represents another central shared mechanism in the pathogenesis of both NAFLD and AF [38]. In NAFLD, insulin resistance increases portal vein concentrations of long-chain fatty acids, while in AF, it has been associated with myocardial inflammation and autonomic dysfunction [38]. Metabolic disturbances characteristic of NAFLD may therefore directly affect atrial conduction properties [35]. Additionally, activation of the renin–angiotensin–aldosterone system contributes both to NAFLD progression—via increased serum free fatty acids and triglycerides—and to myocardial fibrosis [38].

The presence of advanced liver fibrosis further amplifies AF risk. Several studies have demonstrated that NAFLD accompanied by significant fibrosis is associated with a markedly increased incidence of AF [40,41]. Notably, AF risk appears elevated even in younger individuals (20–39 years) with NAFLD and advanced fibrosis [42]. The fatty liver index has therefore been proposed as a screening tool for AF risk in young patients with NAFLD, and elevated values have been associated with incident AF even in apparently healthy populations [28,43,44]. Although NAFLD increases both prevalent and incident AF risk, this association appears stronger in diabetic compared with non-diabetic individuals [45].

Inflammatory and metabolic biomarkers have also been investigated as mediators of this association. An increased monocyte-to-HDL-C ratio has been linked to heightened inflammatory activity and increased AF risk in patients with NAFLD, with a proposed cutoff value of 0.44 [45,46]. HDL-C exerts anti-inflammatory effects by inhibiting monocyte activation and proliferation; thus, the combination of monocytosis and low HDL-C may synergistically promote atrial myocyte dysfunction and AF development [46]. Similarly, an elevated uric acid–to–HDL-C ratio has emerged as another metabolic-inflammatory marker predictive of AF in NAFLD [47].

In a meta-analysis, MASLD was shown to be significantly associated with AF prevalence, although no statistically significant association with incident AF was identified [48]. Hepatic steatosis, inflammation, and fibrosis appear to underlie atrial remodeling processes in MASLD [49,50]. Altered left atrial strain, secondary to increased atrial volume and fibrosis, may facilitate electrophysiological remodeling and the formation of low-voltage atrial areas, thereby promoting AF [49,50]. In patients with MASLD, higher liver fibrosis scores have been associated with more pronounced atrial remodeling and increased AF recurrence following catheter ablation [49]. Patients with severe fibrosis exhibit impaired left atrial compliance, atrial dilation, and reduced contractility, while histopathological analyses reveal an approximately 2.78-fold increase in atrial fibrosis compared with individuals without MASLD [49]. Correspondingly, AF recurrence rates after ablation reach approximately 77% in this population [49]. Other studies report a threefold higher recurrence rate in patients with NAFLD and advanced fibrosis compared with those without fibrosis or with early-stage disease [51]. The relationship between atrial fibrosis, asymptomatic AF burden, and post-ablation recurrence warrants further investigation [52,53].

Importantly, a meta-analysis demonstrated that the association between MASLD and AF remains significant even after adjustment for hypertension, type 2 diabetes mellitus, and body mass index [54]. MASLD promotes both hepatic and systemic insulin resistance, leading to increased release of proinflammatory, profibrotic, prooxidant, and prothrombotic mediators that affect myocardial electrophysiology and accelerate coronary atherosclerosis [54,55]. Increased very-low-density lipoprotein secretion may further contribute to atrial remodeling and atherosclerotic progression [55]. Excess intracellular lipid accumulation within atrial myocytes can damage sarcomeric proteins and disrupt electrophysiological properties, thereby facilitating AF development [55].

The incidence of AF is increased across all histological stages of MASLD, including early non-fibrotic disease [56]. However, data from a Swedish cohort indicate that advanced fibrotic and cirrhotic stages carry the highest risk of incident arrhythmias, including AF, with up to a 12-fold increased risk compared with control populations [57]. In cirrhosis, systemic vasodilation and hyperdynamic circulation—features of cirrhotic cardiomyopathy—further predispose to AF [58]. Intestinal dysbiosis and bacterial translocation resulting from portal hypertension contribute to systemic inflammation, arterial vasodilation, and circulatory dysfunction [58]. Increased hepatic vascular resistance leads to elevated preload and right ventricular afterload, favoring the development of heart failure with preserved ejection fraction and further increasing AF susceptibility [59].

Obesity in the context of MASLD has generated ongoing debate regarding its impact on cardiovascular risk and all-cause mortality, particularly when comparing lean and obese patients [60]. Several studies indicate that the increased risk of cardiovascular, renal, and hepatic events in these populations is mediated by complex pathophysiological mechanisms, including hepatic insulin resistance, systemic inflammation, dyslipidemia, and endothelial dysfunction, potentially superimposed on an immunogenetic predisposition [60]. This subgroup has been described as exhibiting a “normal-weight obese” metabolic phenotype [59]. In a large retrospective analysis of hospitalized patients with metabolic dysfunction-associated steatohepatitis (MASH), the prevalence of AF or atrial flutter was similar in lean (13.0%) and non-lean (12.5%) individuals, suggesting that adiposity alone may not fully explain arrhythmic risk [60].

Genetic studies further support the role of body mass index (BMI) in AF susceptibility, as attenuation of BMI-related genetic risk scores has been associated with reduced AF risk, highlighting the potential benefit of weight reduction strategies [61]. In a large Asian cohort, the risk of AF was increased in non-obese patients with NAFLD diagnosed using the fatty liver index (FLI) [62]. Although AF incidence remains higher in obese individuals, non-obese patients with NAFLD and accompanying components of metabolic syndrome also demonstrate an elevated risk of incident AF [62]. In a cohort of predominantly healthy older adults, liver disease activity assessed by iron-corrected T1 mapping on magnetic resonance imaging and male sex were independently associated with new-onset AF (hazard ratio 1.3) [63]. Patients who experienced major cardiovascular events during a median follow-up of 2.5 years were older, predominantly male, and had higher BMI values [63].

Weight loss has consistently been shown to confer multiple cardiovascular benefits, including improved maintenance of sinus rhythm and reduced AF risk [64]. These effects are mediated through improvements in metabolic syndrome components, blood pressure control, glycemic regulation, lipid profiles, and systemic inflammatory markers [64]. Furthermore, weight reduction is associated with favorable structural cardiac remodeling, including reductions in left atrial volume, left ventricular end-diastolic volume, ventricular wall thickness, and improvements in diastolic function parameters [64]. Despite the recognized association between AF, obesity, and height, specific threshold values that would justify AF screening based on anthropometric parameters, as well as their interaction with age-related risk, remain undefined [65]. A BMI ≥ 30 kg/m^2^ is strongly associated with AF, and genetic variants linked to elevated BMI further increase AF incidence [66].

Multiple studies have also established a relationship between liver cirrhosis and the risk of incident AF [67,68]. Reported AF prevalence in cirrhotic populations varies widely, ranging from 0.2% to 20.2% [69,70,71]. In a large cohort of hospitalized patients with end-stage liver disease, AF prevalence was 7.48%, increasing from 5.73% to 9.75% between 2003 and 2014, possibly reflecting improved diagnostic awareness and surveillance [72]. In this population, advanced age and traditional AF risk factors predominated, with dyslipidemia, prior stroke, and coronary artery disease being the most common comorbidities [72]. Conversely, another study reported an AF prevalence of 6.6% in patients with cirrhosis, comparable to that of the general population, suggesting that cirrhosis per se may not independently increase AF risk [68]. In that cohort, AF was more closely associated with comorbid conditions such as hypertension, diabetes mellitus, chronic kidney disease, chronic lung disease, and vascular disease [68].

In contrast, other authors have reported AF as the most frequent arrhythmia in cirrhotic patients, occurring in up to 37.3% of cases [73]. Beyond classical AF mechanisms, cirrhosis introduces additional proarrhythmogenic factors, including left atrial enlargement, atrial interstitial fibrosis, hepatorenal syndrome, electrolyte and metabolic disturbances, and elevated serum bile acid levels [74]. Conversely, systemic hypotension and downregulation of myocardial β-adrenergic receptors may exert a protective effect, potentially reducing AF prevalence in some cirrhotic patients [74]. Pharmacological therapies commonly used in cirrhosis, such as anti-aldosterone diuretics, angiotensin-converting enzyme inhibitors, beta-blockers, and statins, may further contribute to this effect [74].

Advanced liver disease, particularly cirrhosis associated with NAFLD, has been shown to carry a higher AF prevalence compared with non-cirrhotic NAFLD [75]. This has been attributed to older age and more advanced fibrosis in cirrhotic patients, both of which independently increase AF risk [75]. Hypoalbuminemia has also been associated with increased AF prevalence, reflecting advanced liver fibrosis and heightened systemic inflammation mediated by cytokines such as interleukin-6 and tumor necrosis factor [76].

Additional studies have identified age and the presence of ascites as independent predictors of AF in patients with cirrhosis [77]. Critically ill cirrhotic patients exhibit an AF prevalence of approximately 14.18%, with AF serving as an independent predictor of all-cause mortality [78]. In decompensated cirrhosis, systematic screening for AF may therefore be clinically relevant for both preventive and therapeutic strategies. Galectin-3, a profibrotic and proinflammatory biomarker elevated in advanced liver disease, has demonstrated predictive value for paroxysmal AF [79,80].

Finally, the coexistence of cirrhotic cardiomyopathy and hepatorenal syndrome has led to the conceptualization of a hepatocardiorenal syndrome, in which systemic inflammation, endothelial dysfunction, and neurohormonal activation play central roles in cardiovascular instability and arrhythmogenesis [81].

Several studies have reported an association between increased liver stiffness, assessed by elastography, and incident AF, with an incidence rate of approximately 10.2 per 1000 person-years [82,83]. Increased liver stiffness has also been linked to enlargement of the inferior vena cava and hepatic veins, suggesting that venous congestion may act both as a cause and a consequence of hepatic stiffness, even in the absence of overt heart failure [83]. Notably, after adjustment for conventional risk factors—including hypertension, diabetes mellitus, dyslipidemia, heart disease, and waist circumference—no significant association was found between AF (prevalent or incident) and hepatic steatosis, NAFLD, or MAFLD in some cohorts, a finding partially attributed to a lower mean BMI in the studied population [83].

Several non-invasive indices are currently used to assess liver fibrosis. The Fibrosis-4 (FIB-4) index has been shown to be useful not only for estimating liver damage but also for predicting cardiovascular events in patients with non-valvular AF [84]. In patients with AF, no universal FIB-4 cut-off has been established, as its components—aspartate aminotransferase (AST), alanine aminotransferase (ALT), platelet count, and age—are themselves markers of liver fibrosis [84]. However, a FIB-4 value > 2.51 has been identified as an independent predictor of adverse cardiovascular outcomes and all-cause mortality, including among patients with paroxysmal AF, particularly in those with a CHA_2_DS_2_-VASc score ≥ 2 [84]. Proteomic analyses further suggest that the relationship between FIB-4 and inflammation predicts prevalent—but not incident—AF [85].

Genetic susceptibility may also underlie the shared inflammatory and immune pathways observed in NAFLD and AF. Some authors have identified eight common hub genes implicated in both diseases, while others advocate for genetic testing in patients with structural heart disease, given the complex polygenic architecture of AF and its frequent comorbidities [86,87].

Beyond liver pathology, other gastrointestinal disorders have been associated with AF. Gastroesophageal reflux disease may trigger AF via parasympathetic activation and esophageal inflammation, modulating atrial refractoriness and inducing vagally mediated ischemia [88]. Gastrointestinal malignancies—particularly esophageal and colorectal cancers—have also been linked to AF through multiple mechanisms, including systemic inflammation, electrolyte imbalance, thrombogenicity, autonomic nervous system activation, and, in some cases, direct mechanical effects on the atria [88,89]. Large hiatal hernias can exert extrinsic compression on the left atrium or pulmonary veins, potentially inducing AF even in younger individuals, as described in gastro-cardiac (Roemheld) syndrome [90,91].

Moreover, gut microbiota dysbiosis has been proposed as an additional pathophysiological pathway linking liver disease and atrial fibrillation. In patients with cirrhosis and metabolic dysfunction-associated steatotic liver disease, intestinal dysbiosis—characterized by reduced *Bifidobacterium* and *Firmicutes* species and increased *Bacteroides* and *Enterococcaceae*—promotes bacterial translocation and lipopolysaccharide-mediated endotoxemia [80]. These processes contribute to systemic inflammation, activation of NLRP3 inflammasomes, impaired glucose tolerance, accumulation of metabolites such as trimethylamine N-oxide, short-chain fatty acids, and indoxyl sulfate, as well as autonomic nervous system dysregulation [80,88,92]. Collectively, these mechanisms may facilitate atrial fibrosis and electrophysiological remodeling, thereby increasing susceptibility to atrial fibrillation [92].

Artificial intelligence has emerged as a powerful tool for early AF detection—even during sinus rhythm on standard electrocardiograms—as well as for risk stratification and personalized management strategies [93]. In the setting of liver disease, artificial intelligence-based methodologies may be particularly advantageous, as patients with MASLD and advanced cirrhosis frequently present with non-classical clinical features, including autonomic dysregulation, systemic inflammation, and complex metabolic alterations that may affect atrial electrophysiological properties [93,94,95,96,97]. In this context, AI-driven electrocardiographic and multimodal analytical frameworks have the potential to facilitate the earlier identification of subclinical AF and to enhance risk stratification along the liver–heart continuum, particularly in patient populations in whom conventional cardiovascular risk scores may inadequately capture arrhythmic vulnerability [93,94,95,96,97].

In the present study, epidemiological analysis indicates that liver pathology is not an incidental finding but a defining characteristic of the population with paroxysmal AF (Figure 4 and Figure 5). Given the retrospective and cross-sectional nature of our cohort, these findings should be interpreted as demonstrating a strong clinical association rather than a direct causal relationship between liver disease and AF. Nearly two thirds of patients (65.2%) exhibited some form of liver disease, most commonly hepatic steatosis (46.2%). This high prevalence is consistent with prior observational and population-based studies reporting a strong association between AF and MASLD, particularly in cohorts characterized by a high cardiometabolic burden. Moreover, this pattern mirrors previous reports describing sex-related differences in liver disease progression, with male patients more frequently exhibiting advanced fibrotic and cirrhotic stages.

Although the overall prevalence of liver disease was similar between sexes (*p* ≈ 0.81), subtle differences were observed: women more frequently exhibited hepatic steatosis, whereas men showed a higher prevalence of cirrhosis. While these differences did not reach statistical significance, they are in line with prior observations suggesting sex-specific disease trajectories, with male patients more prone to progression toward advanced fibrotic stages and cirrhosis, potentially reflecting differences in hormonal, metabolic, and lifestyle-related risk modifiers reported in the literature [75,76].

AF is strongly age-dependent, and elderly patients commonly present with overlapping cardiovascular and gastrointestinal comorbidities [98] (Figure 1 and Figure 2). To disentangle these associations, multivariate logistic regression was applied, allowing for the identification of independent predictors and revealing two distinct hepatic-AF phenotypes:

A. Metabolic–steatosis phenotype

This profile is characterized by obesity and dyslipidemia as dominant risk factors. The likelihood of hepatic steatosis increased markedly in the presence of dyslipidemia (adjusted OR 4.51; 95% CI 2.23–9.12; *p* < 0.001) and obesity (adjusted OR 2.54; 95% CI 1.15–5.65; *p* = 0.02) [32,33,34,35]. These findings are consistent with existing literature placing dyslipidemia, obesity, and metabolic syndrome at the core of the pathophysiological link between metabolic dysfunction-associated steatotic liver disease, atrial fibrillation, and increased cardiovascular risk [32,33,34,35].

B. Cirrhotic phenotype (lipid paradox)

In contrast, dyslipidemia was inversely associated with liver cirrhosis (adjusted OR 0.14; 95% CI 0.03–0.63; *p* = 0.01), reflecting a phenomenon commonly referred to as the “lipid paradox” [36,37,38,39]. This term describes the paradoxically normal or low serum lipid levels observed in advanced cirrhosis, which result from impaired hepatic lipid synthesis and severe hepatocellular dysfunction rather than from a protective metabolic profile. Accordingly, this inverse association does not indicate reduced cardiovascular risk but instead reflects advanced liver failure, with diminished cholesterol and triglyceride production—a pattern previously reported in patients with cirrhosis and elevated cardiovascular risk [36,37,38,39]. Clinically, patients within this cirrhotic phenotype often present at younger ages, likely due to premature mortality, and may display deceptively favorable lipid profiles despite carrying the highest overall risk, particularly when atrial fibrillation coexists with advanced liver disease.

In this context, anticoagulation therapy in patients with cirrhosis and atrial fibrillation poses substantial clinical challenges. Thrombocytopenic hypersplenism represents a key complicating factor, increasing bleeding risk and limiting therapeutic options. In selected cases, platelet count improvement through minimally invasive interventions, such as partial splenic embolization, has been validated as a potential therapeutic strategy [98,99].

### Study Design Limitations

This study has inherent limitations related to its retrospective design and the inclusion of patients admitted over a 10-year period to a tertiary emergency hospital. Paroxysmal AF was electrocardiographically documented; however, the timing of AF onset and echocardiographic data were unavailable in many records. Hepatic steatosis was primarily diagnosed by ultrasonography, while patients with neoplastic liver disease underwent additional multimodal imaging, including contrast-enhanced computed tomography (CT) and/or magnetic resonance imaging (MRI).

Although the overall sample size of 253 patients provided adequate statistical sensitivity for analyses of prevalent conditions such as hepatic steatosis, the subgroup with cirrhosis was smaller (*n* = 45), which may have limited the ability to detect weak associations in this subgroup. Nevertheless, the principal findings observed in patients with cirrhosis—particularly the strong inverse association with dyslipidemia (OR = 0.14)—exceeded the minimum detectable effect size derived from sensitivity analyses. The magnitude of this association suggests a robust biological signal that persists despite the smaller number of events and supports the validity of the reported results.

It should be emphasized that the hepatic phenotypes described in this study were defined a priori and are supported by multivariable logistic regression analyses, rather than derived from unsupervised approaches such as cluster analysis. Accordingly, the term “phenotype” is used to denote clinically and metabolically distinct profiles identified through adjusted association patterns. Formal validation using dedicated classification methods, including cluster analysis or discriminant modeling, will require larger cohorts with more comprehensive metabolic and imaging data.

Consistent with the retrospective nature of the study, information regarding anticoagulant therapy (type, dosage, duration, adherence, and temporal changes) was not documented uniformly and was not available in a standardized format for all patients. Consequently, anticoagulation could not be included as an adjustment variable, and residual confounding cannot be excluded. In addition, alcohol consumption was recorded only as a qualitative, dichotomous variable (present/absent), without standardized quantification of exposure (i.e., degree of alcohol consumption). Furthermore, no validated comorbidity severity index (such as the Charlson or Elixhauser Comorbidity Index) was available; therefore, overall comorbidity burden was described using the total number of recorded diagnoses per patient (“No. Diag.”), an approach that carries inherent limitations.

## 5. Conclusions

Liver pathology exhibits a significant prevalence among individuals diagnosed with paroxysmal AF, impacting nearly two-thirds of the studied cohort, and constitutes an essential component of their clinical and metabolic risk assessment. In this context, two distinct hepatic phenotypes are identified: a metabolic–steatosis phenotype correlated with dyslipidemia and obesity, and an advanced cirrhotic phenotype distinguished by low lipid levels and a younger demographic, reflecting disease severity rather than metabolic protection. Venous congestion, ascites-related endotoxemia, neurohormonal activation, hepatorenal syndrome, electrolyte disturbances, gut microbiota dysbiosis, systemic inflammation, and cirrhotic cardiomyopathy represent interrelated pathophysiological mechanisms that may facilitate AF in advanced liver disease.

Individuals suffering from AF and liver involvement display a more unfavorable metabolic and lifestyle profile, including higher rates of diabetes, dyslipidemia, obesity, and alcohol consumption. These findings underscore the importance of systematic hepatic and metabolic evaluation in patients with AF and suggest that integrated, multidisciplinary management may improve risk stratification and therapeutic decision-making.

## Figures and Tables

**Table 1:** Major comorbidity categories and included diagnoses in patients with paroxysmal atrial fibrillation.

Comorbidity Category	Included Conditions
Cardiovascular	Arterial hypertension, coronary artery disease, chronic heart failure, valvular heart disease, stroke, conduction disorders, pacemaker implantation
Hepatic and portal	Hepatic steatosis, liver cirrhosis, chronic liver failure, ascites, esophageal varices, primary and secondary liver tumors
Metabolic/endocrine	Type 2 diabetes mellitus, dyslipidemia, obesity, hypothyroidism
Non-hepatic digestive	Gastroesophageal reflux disease, diverticulosis, gastrointestinal bleeding, colonic polyps
Renal	Chronic kidney disease, benign renal tumors
Respiratory	Chronic obstructive pulmonary disease, asthma, pulmonary fibrosis, obstructive sleep apnea
Neoplastic	Digestive, pulmonary, urological, breast, thyroid, hematological malignancies
Other systemic/infectious	Anemia, sepsis, COVID-19, autoimmune disorders
Lifestyle factors	Alcohol consumption, smoking

**Table 2:** Serum biomarkers across hepatic phenotypes (median [Q1–Q3], N; Kruskal–Wallis *p* value).

Parameter	No Liver Disease	Hepatic Steatosis	Advanced Liver Disease/Cirrhosis	*p* (Kruskal–Wallis)
ALT (U/L)	18.20 [10.00–34.90], N = 31	22.20 [16.30–43.20], N = 45	26.60 [14.07–41.35], N = 22	0.39681
AST (U/L)	19.10 [13.60–45.95], N = 31	23.00 [18.00–30.95], N = 47	31.70 [20.80–79.00], N = 21	0.02153
Albumin	3.64 [2.81–4.03], N = 16	3.76 [3.44–4.36], N = 30	3.23 [2.54–3.79], N = 18	0.01052
Total bilirubin	0.44 [0.33–0.62], N = 35	0.67 [0.40–0.86], N = 50	0.95 [0.56–1.83], N = 21	0.00177
Alkaline phosphatase (ALP)	77.00 [53.50–92.00], N = 18	66.00 [56.00–97.00], N = 35	100.00 [74.50–115.25], N = 14	0.02532
Cholesterol	138.40 [120.90–158.20], N = 29	177.00 [132.55–207.80], N = 40	115.85 [80.70–153.90], N = 20	0.00118
Triglycerides	104.95 [66.92–145.25], N = 24	95.75 [78.58–150.18], N = 36	87.80 [62.32–103.95], N = 18	0.30758
INR	1.34 [1.12–2.09], N = 32	1.12 [1.04–1.20], N = 50	1.61 [1.28–2.47], N = 26	0.00001
Platelets (/µL)	242,500 [190,500–327,750], N = 38	239,000 [193,500–317,250], N = 54	166,000 [106,500–252,500], N = 27	0.00318
Hemoglobin	9.00 [6.72–12.65], N = 39	12.70 [10.08–14.00], N = 54	10.70 [8.85–12.45], N = 27	0.00154

## Data Availability

The original contributions presented in this study are included in the article. Further inquiries can be directed to the corresponding author.

## Abbreviations

The following abbreviations are used in this manuscript:

AF	Atrial fibrillation
AI	Artificial intelligence
ALT	Alanine aminotransferase
AST	Aspartate aminotransferase
BMI	Body mass index
BP	Blood pressure
CHA_2_DS_2_-VASc	Stroke risk score (Congestive heart failure, Hypertension, Age ≥ 75, Diabetes, Stroke/TIA, Vascular disease, Age 65–74, Sex category)
CI	Confidence interval
COPD	Chronic obstructive pulmonary disease
COVID-19	Coronavirus disease 2019
CT	Computed tomography
DRG	Diagnosis-Related Group
EPV	Events per variable
FIB-4	Fibrosis-4 index
FLI	Fatty liver index
GERD	Gastroesophageal reflux disease
HDL-C	High-density lipoprotein cholesterol
IL	Illinois (in the affiliation “Chicago, IL, USA”)
MASH	Metabolic dysfunction-associated steatohepatitis
MASLD	Metabolic dysfunction-associated steatotic liver disease
MeSH	Medical Subject Headings
MRI	Magnetic resonance imaging
NAFLD	Nonalcoholic fatty liver disease
NLRP3	NLR family pyrin domain containing 3 (inflammasome)
OR	Odds ratio
*p*	*p* value
RR	Risk ratio
SPSS	Statistical Package for the Social Sciences
TNF-α	Tumor necrosis factor alpha
USA	United States of America
α	Alpha (significance level)
χ^2^	Chi-square (test)
